# Bridging gaps: On the performance of airborne LiDAR to model wood mouse-habitat structure relationships in pine forests

**DOI:** 10.1371/journal.pone.0182451

**Published:** 2017-08-03

**Authors:** Carlos Jaime-González, Pablo Acebes, Ana Mateos, Eduardo T. Mezquida

**Affiliations:** Department of Ecology, Faculty of Sciences, Universidad Autónoma de Madrid, Madrid, Spain; University of Maine, UNITED STATES

## Abstract

LiDAR technology has firmly contributed to strengthen the knowledge of habitat structure-wildlife relationships, though there is an evident bias towards flying vertebrates. To bridge this gap, we investigated and compared the performance of LiDAR and field data to model habitat preferences of wood mouse (*Apodemus sylvaticus*) in a Mediterranean high mountain pine forest (*Pinus sylvestris*). We recorded nine field and 13 LiDAR variables that were summarized by means of Principal Component Analyses (PCA). We then analyzed wood mouse’s habitat preferences using three different models based on: (i) field PCs predictors, (ii) LiDAR PCs predictors; and (iii) both set of predictors in a combined model, including a variance partitioning analysis. Elevation was also included as a predictor in the three models. Our results indicate that LiDAR derived variables were better predictors than field-based variables. The model combining both data sets slightly improved the predictive power of the model. Field derived variables indicated that wood mouse was positively influenced by the gradient of increasing shrub cover and negatively affected by elevation. Regarding LiDAR data, two LiDAR PCs, i.e. gradients in canopy openness and complexity in forest vertical structure positively influenced wood mouse, although elevation interacted negatively with the complexity in vertical structure, indicating wood mouse’s preferences for plots with lower elevations but with complex forest vertical structure. The combined model was similar to the LiDAR-based model and included the gradient of shrub cover measured in the field. Variance partitioning showed that LiDAR-based variables, together with elevation, were the most important predictors and that part of the variation explained by shrub cover was shared. LiDAR derived variables were good surrogates of environmental characteristics explaining habitat preferences by the wood mouse. Our LiDAR metrics represented structural features of the forest patch, such as the presence and cover of shrubs, as well as other characteristics likely including time since perturbation, food availability and predation risk. Our results suggest that LiDAR is a promising technology for further exploring habitat preferences by small mammal communities.

## Introduction

Forest ecosystems and the biodiversity they hold are facing increasing pressures due to natural factors and management practices that alter and, in many cases, simplify habitat heterogeneity [1]. These *a priori* complex habitats are mainly determined by vegetation structure, which is widely recognized as being one of the most important factors in habitat selection for numerous taxonomic groups [2]. Among them, birds are by far the most investigated taxa after the seminal study of MacArthur and MacArthur [3] linking bird species diversity to canopy vertical heterogeneity.

Vegetation structure is commonly described by canopy cover/openness, canopy height, canopy vertical complexity or biomass [4, 5]. Until the advent of remote sensing technologies such as Light Detection and Ranging (LiDAR), this type of fine-scaled variables was largely restricted to observational field surveys, which are often laborious, difficult to measure and subjective by an observer on the ground [6–8]. For that reason, LiDAR technology has brought back with renewed strength the classical study of MacArthur and MacArthur [3], while it has contributed to revitalize the “habitat heterogeneity hypothesis” (structurally complex habitats can provide more niches and thus increase species diversity; [2]) by providing accurate and objective measures of vegetation architecture to model wildlife-habitat structure relationships. A growing list of LiDAR-based studies has been published during the last decade, although studies on three-dimensional (3-D) animal ecology are strongly biased toward flying vertebrates, mainly birds (but also bats), given the 3D nature of bird ecology [9, 10]. The most relevant predictor regarding vegetation structure for flying vertebrates was canopy vertical heterogeneity, as it leads to an increase in available niches (see review by [9]). The paucity of studies of nonflying vertebrates in forests probably has to do with not-so-obvious relevance of habitat structure for these other taxa (except for tree-dwelling squirrels and primates; [11, 12]). Nevertheless, canopy cover and understory vegetation derived from LiDAR data have been shown to influence hunting or foraging decisions and habitat use under contrasting weather conditions in some terrestrial mammals such as ungulates or meso-carnivores [9, 13–15].

One group for which LiDAR data has not been applied is ground-dwelling small rodents (hereinafter small mammals). This heterogeneous group play important roles in the dynamics of forest ecosystems, acting, for example, as seed and seedling predators, but also as dispersers of seeds and spores of plants and fungi, or contributing to organic matter decomposition and soil mixing [16, 17]. Further, they are important food resources for other species, comprising the main prey for many avian and terrestrial predators [18, 19].

In this study, we use wood mouse (*Apodemus sylvaticus*) as the target species. The wood mouse is a common scatter-hoarding rodent in Mediterranean habitats [20], and probably the most abundant mammal in forest systems in the Iberian Peninsula due to its generalist character and ability to adapt to different environments [21]. Previous research has examined wood mouse’s habitat preferences in forests and assessed the role of different elements such as the amount of coarse woody debris, litter or understory vegetation and the age, structure or heterogeneity of forests stands [22–27]. These studies (based on field measurements) found that these factors influenced the abundance of this species through their effects on food provision and availability of nesting sites and shelter from predators [28]. In this regard, Airborne LiDAR data may be a powerful tool used in studies on small mammals, provided it can accurately capture habitat features important for this ground-dwelling species. For example, characterization of lower layers using LiDAR data may be less precise in closed habitats dominated by perennial trees, such as pine forests (e.g. [29–31]). However, the accuracy of LiDAR derived variables has been broadly demonstrated in early applications to forest inventory and later use in ecological research [9, 10].

Given that the majority of published literature using LiDAR investigate bird responses to habitat structure [9, 10], this study aimed to narrow this gap by providing new insights on small mammals-habitat structure relationships through the combined use of field and LiDAR derived environmental variables in a Mediterranean high mountain pine forest. Our first objective was to assess the value of airborne LiDAR to model wood mouse habitat preferences. Our second objective was to evaluate the performance of traditional field-based variables to explain wood mouse preferences. Finally, we combined LiDAR- and field-based variables in the same predictive model to determine whether the predictive power of the model increased compared to models using one dataset. Moreover, we used variance partitioning to assess the independent and joint contribution of each type of explanatory variables. To our knowledge, this is the first attempt to model habitat preferences of a small mammal using LiDAR data.

We hypothesized that habitat features measured in the field such as shrub and litter cover would better explain wood mouse habitat preferences rather than LiDAR-based measures, since (i) this type of fine-scale microhabitat variables has been previously described as relevant for small mammals; (ii) previous studies have shown the difficulty of obtaining accurate understory LiDAR-based measures in closed-canopy forests of perennial-dominated species, and (iii) small non-flying vertebrates do not seem to be directly affected by canopy cover or vertical structure of the canopy.

## Materials and methods

### Study area

The study was conducted in the forest of Valsaín (7622 ha), located on the northern slope of the Guadarrama Mountain Range, central Spain (40° 51´N, 4° 3´W). The forest is mostly dominated by Scots pine (*Pinus sylvestris*), a typically boreal species, mainly restricted in southern Europe to the high mountains of the Mediterranean basin [32], with Pyrenean oak (*Quercus pyrenaica*) being more abundant at lower elevations. The shrub layer is mainly comprised by *Genista florida*, *Cytisus scoparius*, *Juniperus communis*, *Rosa* sp., *Rubus* sp. and *Ilex aquifolium*. The pine forest occurs between 1200 m and 2100 m on acidic soils. The climate is continental Mediterranean, with hot, dry summers and cold winters, with mean precipitation ranging from over 900 to 1500 mm, depending on elevation, and mean annual temperature of around 9.8°C.

The management that is carried out in this pine forest consists of the exploitation of timber resources while maintaining natural forest productivity, with a complete reliance on natural regeneration success. Using a group shelterwood method, a new stand is established by gradually removing all trees in a series of repeated partial harvests over a rotation period of 120 years. To do so, thinning of the tree mass is made, combined with the creation of small gaps (0.1–0.2 ha) in the forest. This allows the progressive establishment of natural regeneration under the protection of seed trees, without additional treatments [33].

### Plot setting and live trapping

**Fig 1:**
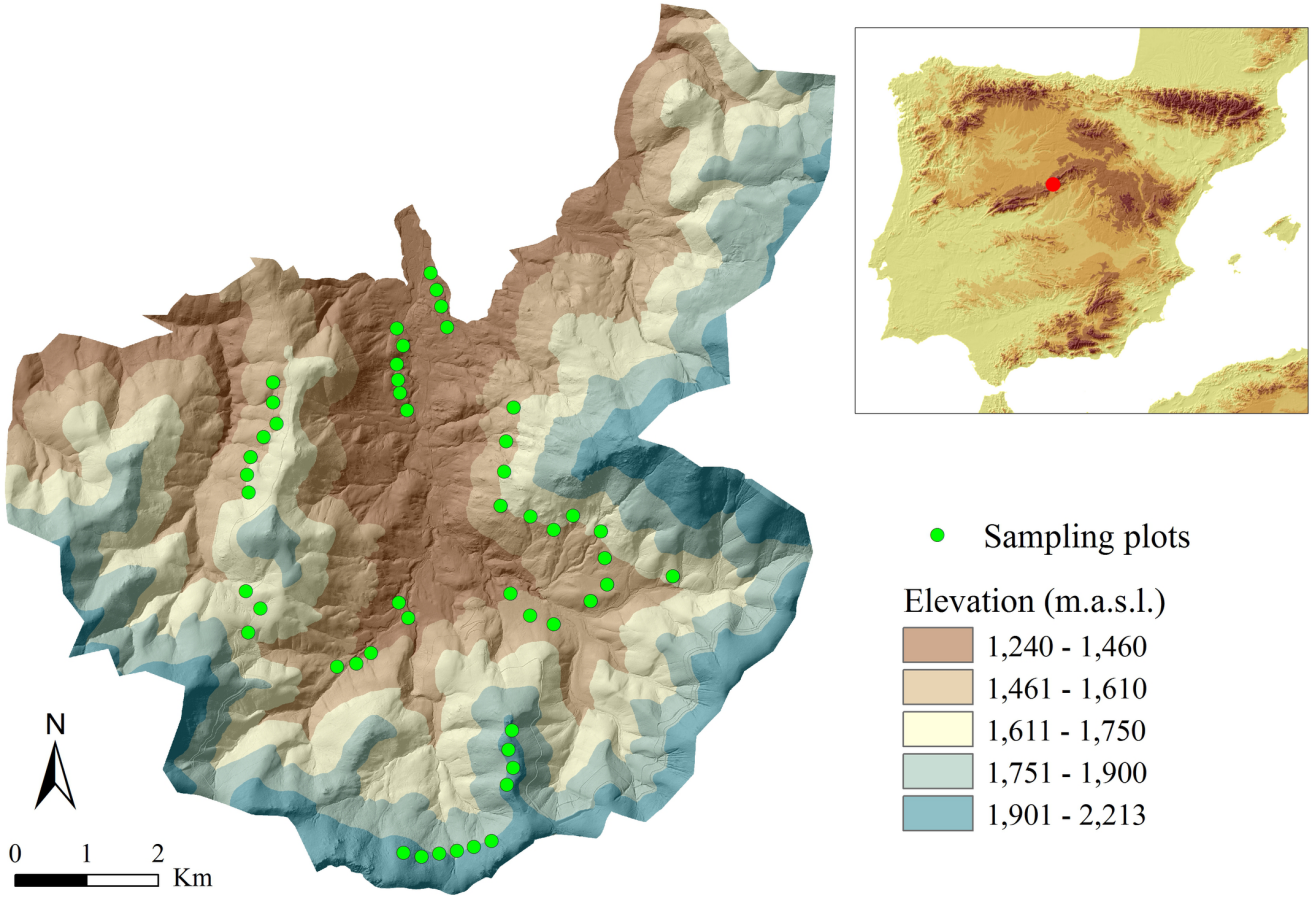
Sampling plots in the pine forest of Valsaín. Location of Valsaín (red dot) in the Central Mountain Range in the Iberian Peninsula, Southern Europe (right). Digital Elevation Model of Valsaín (left) showing the sampling plots (green dots).

Fifty 25 m radius circular plots (0.196 ha) were located in the pine forest of Valsaín along the whole altitudinal range (Fig 1), with a minimum distance of 250 m between them. The separation between plots was aimed to cover a wide gradient of forest structures. At each circular plot, a grid of 16 Sherman traps (20 x 6 x 6 cm) was set up to trap small mammals. Two traps were placed in the direction of each of the four cardinal points and its four diagonals, at a distance of 8 and 16 m from the center of the plot, thus creating two evenly spaced concentric rings of traps. Traps were set as horizontal as possible to increase the likelihood of captures, and covered with leaf litter to improve thermal insulation and to conceal them. Each trap was baited with bread fried in rancid oil and waterproof cotton was added to reduce the risk of hypothermia [34]. Trapping sessions were conducted from mid-June to mid-July 2014, coinciding with an increase in wood mouse activity in Mediterranean high mountain areas [21]. Traps were active during four consecutive nights and were checked early each morning. Total trapping effort was therefore 3200 traps over the four nights.

Trapped individuals were identified to species, sexed, weighed with a digital scale to the nearest 0.1 g and their breeding condition was determined following Gurnell and Flowerdew [35]. Before releasing, each individual was marked with a temporary color code to control for potential recaptures. Manipulation time was kept at minimum to avoid disturbance to animals and each individual was released at its trapping site.

### Ethics statement

Capture and handling of individuals were done in compliance with the Spanish Royal Decree-Law 53/2013 and the European Union Directive 2010/63/EU on the protection of animals used for scientific purposes and were conducted under permit from Servicio Territorial de Medio Ambiente de la Junta de Castilla y León, Spain (Reference: EP/SG/608/2013). The Institutional Animal Care and Use Committee from the Universidad Autónoma de Madrid was prospectively consulted about the need of a specific permit for animal trapping and handling. Captures did not involve endangered nor protected species and trapping and handling did not include invasive procedures or others that could negatively affect animal welfare, thus a specific permit was not necessary.

### Field-based understory composition and structure

At each of the 50 plots, 8–11 2 x 2 m quadrats were set up next to the live traps sited in the four cardinal directions or its diagonals, for a total of 446 quadrats measured. In each quadrat, we visually estimated the percent cover of shrubs and small trees (*G*. *florida*, *C*. *scoparius*, *J*. *communis*, *Rosa* sp., *Rubus* sp. and *I*. *aquifolium*), forbs, ferns (*Pteridium aquilinum*), mosses, needle litter, woody debris and bare rock. We also measured the average height of shrubs and ferns using a metric tape. Cover variables were estimated independently for each species, so total cover may exceed 100% [36]. We finally calculated the mean for each variable to obtain average values per plot.

### LiDAR-based forest structure

**Fig 2:**
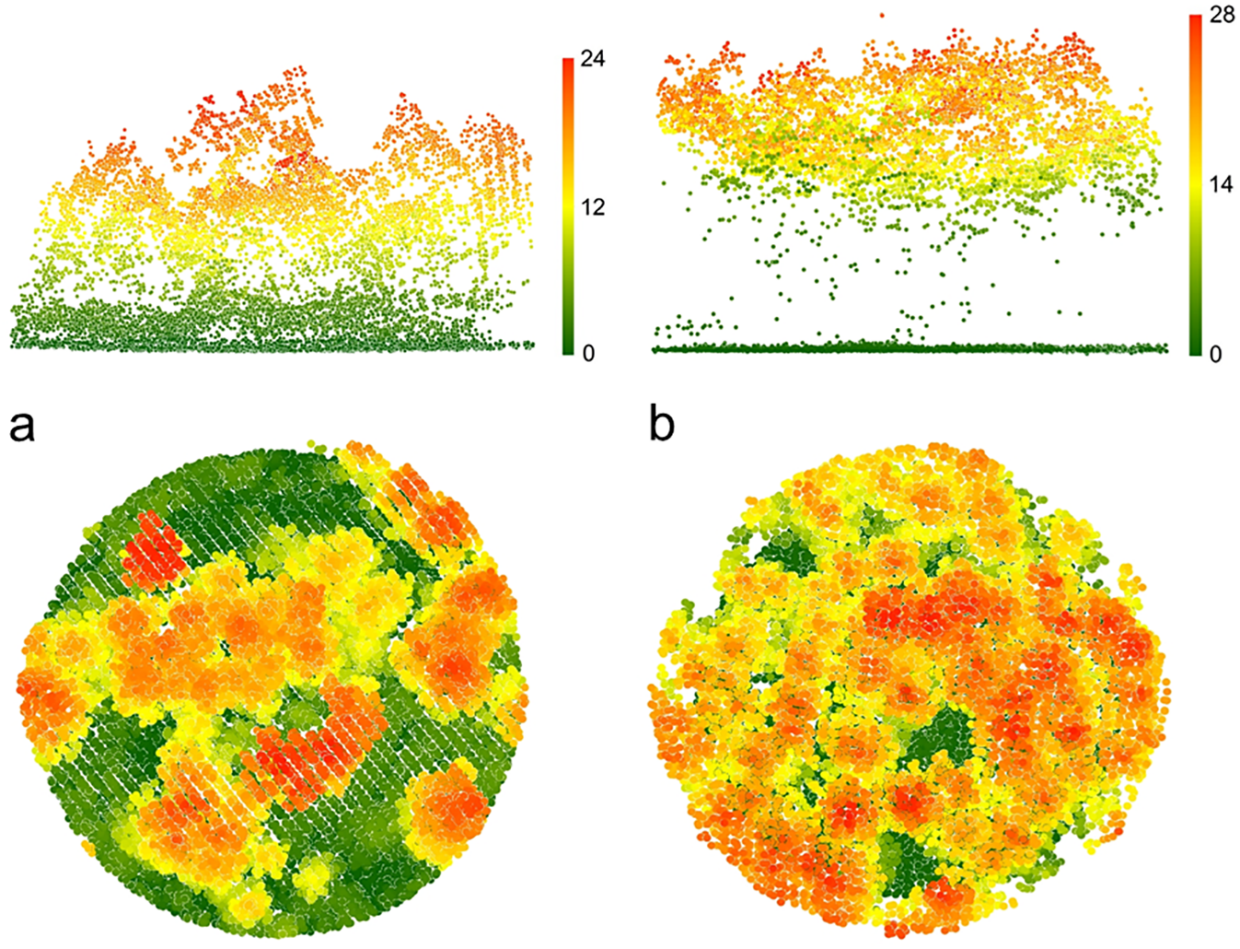
Elevation and plan illustrations of two plots situated at opposing extremes of the structural gradient. (a) plot with an open structure, consisting of tree and regeneration layers and well developed understory, in which individuals of *A*. *sylvaticus* were captured. (b) plot with a closed canopy cover and with an absence of understory vegetation, in which no small mammals were captured. The colour gradient represents the variation in height (m) of the forest structure obtained from the LiDAR point data.

Forest structure variables (horizontal and vertical) at the plot level were derived from LiDAR data collected in a Piper PA31 Navajo aircraft provided by Blom Sistemas Geoespaciales, S.L.U in 2009. The LiDAR sensor used was a Leica ALS 60 with a pulse density between 4.7 and 7.3 pulses m^2^. Flight speed and altitude were 85 m/s and 891 m respectively. The processing of the raw data from the LiDAR point cloud was performed with the software FUSION ([37]; Fig 2).

Vegetation 3D structure was assessed by thirteen variables. These variables were grouped into four categories, that informed us about (i) canopy cover, (ii) composition of vertical structure, (iii) heterogeneity of vertical structure, and (iv) canopy mean height. The horizontal structure was estimated from the percentage of first laser returns in the LiDAR point cloud at 5, 2 and 0.2 m, which is an indicator of canopy cover at each height. Therefore, canopy cover at 5 m indicates the cover of mature trees, whereas canopy cover at 2 m includes the cover of mature trees plus young trees (i.e., the regeneration layer). Canopy cover at 0.2 m includes the latter plus the shrub cover.

The complexity of the vertical structure was estimated for four forest layers: mature tree layer (>5 m), regeneration layer (2–5 m), shrub layer (0.2–2 m) and herbaceous layer (<0.2 m). The contribution of the four layers sums up to 1. Thus, values close to one in the mature tree layer indicate dense tree crowns with limited presence of additional vertical structure at lower layers. On the other hand, values close to zero indicate open canopies with lower layers comprising most of the total vertical structure.

We also calculated the heterogeneity of canopy height for the mature tree layer (>5 m), for the mature plus regeneration layer (>2 m) and for the mature plus regeneration plus shrub layer (>0.2 m), as the standard deviation of height values for first returns up to 5, 2 and 0.2 m, respectively [9]. Finally, the mean height of vegetation was also estimated for tree, regeneration and shrub layers. As the year of small mammal trapping (2014) differed from the acquisition of LiDAR data (2009), the choice of sampling plots was made according to the information provided by forest managers, in order to avoid areas where silvicultural activities had occurred in the intervening 5 years, and this was ground-truthed in the field.

### Statistical analyses

Mean height of shrubs and ferns measured in quadrats were both highly correlated with their corresponding cover variables (*r* > 0.89, in both cases), so we excluded heights from further analyses. Field- and LiDAR-based habitat variables at the plot scale were summarized by two separate principal component analyses (PCA) from their respective correlation matrices due to the high collinearity among the original variables. Varimax rotated components with eigenvalues > 1 were retained in order to extract factors that represented major underlying gradients with ecological meaning, that were then used as predictors in models of habitat preference by wood mice. Because the abundance of small mammals usually decreases with elevation in Mediterranean mountains [38], and elevation may interact with forest structure, we calculated the elevation of each sampling plot from a 5-m resolution digital elevation model of the study area. Plot elevation was standardized to zero mean and unit variance for the analyses.

In order to assess whether wood mouse habitat preferences were better predicted by field- or LiDAR-based variables, we firstly built a generalized linear model for each sampling approach (field *versus* LiDAR data), and secondly a full model including both sets of variables. The response variable for the three models was the number of individuals captured per plot (excluding recaptures). The predictor variables were the components extracted from the PCA of field-based variables, the components extracted from the PCA of LiDAR-based variables or all components for the full model. Plot elevation and the interaction between elevation and each component of the PCA were also included as predictors in the models. A Poisson distribution of residuals was assumed in all models. Initial models were simplified by progressively removing the least significant terms and their goodness of fit were assessed using the Akaike’s Information Criterion (AIC; [39]).

To evaluate the relative importance of the independent versus the shared part of variance explained by the explanatory variables (i.e., field- or LiDAR-based variables and elevation) on wood mice habitat preferences, we used variance partitioning for the best model obtained after simplification of the full model (including both groups of explanatory variables). To do so, we calculated goodness of fit measures (Log-Likelihood) for the entire hierarchy of models (i.e., models including all combinations of independent variables). Then, the hierarchical partitioning algorithm of Chevan and Sutherland [40] was applied taking the list of goodness of fit measures to calculate the independent contribution of each variable and its conjoint contribution with all other variables [41]. All analyses were performed in R 3.1.1 [42] using the stats and the hier.part packages [41].

## Results

### Captures

A total of 225 individuals were captured; nearly all were wood mice (*n* = 223), 56 of which were recaptures, and 2 were Lusitanian pine voles (*Microtus lusitanicus*). The 56 recaptured wood mice and the two Lusitanian pine voles were excluded from further analyses. Wood mice were captured in 42 of the 50 sampling plots, with the number of captures per plot ranging from 1 to 13 individuals. Most of the 167 first capture wood mice could be aged and sexed: 29% were sub-adults, 32% were adult males and 36% were adult females; the remaining 3% were of unknown age or sex. More than half of adult females (72%) showed signs of breeding activity.

### Field-based understory composition and structure

Understory field-based variables presented a considerable degree of variation, with most showing some percentage cover of forbs and needle litter, and a patchy cover of shrubs, ferns and mosses, ranging from zero to 58, 64 and 36%, respectively (Table 1).

**Table 1:** Summary of field and LiDAR-based variables at plot scale (25-m radius).

Variables	Method	Range	Mean	SD
Shrub cover	Field	0–58.3	9.3	11.5
Forb cover	Field	5.3–83.7	36.0	16.6
Fern cover	Field	0–64.4	13.1	16.8
Moss cover	Field	0–36.2	9.1	11.1
Needle litter cover	Field	5.8–80.6	38.4	15.8
Woody debris cover	Field	9.4–38.5	20.6	6.8
Bare rock cover	Field	0–39.4	9.8	8.6
Canopy cover at 5 m	LiDAR	38.9–94.0	70.1	14.3
Canopy cover at 2 m	LiDAR	47.2–94.1	74.9	11.9
Canopy cover at 0.2 m	LiDAR	62.0–94.6	80.1	9.4
Contribution of the tree layer	LiDAR	36.8–75.3	57.8	10.1
Contribution of the regeneration layer	LiDAR	0.1–19.1	4.8	4.7
Contribution of the shrub layer	LiDAR	0.5–18.3	6.2	4.5
Contribution of the herbaceous layer	LiDAR	16.0–45.6	31.1	7.4
Heterogeneity of the canopy height (>5 m)	LiDAR	1.5–8.2	4.1	1.6
Heterogeneity of the canopy height (>2 m)	LiDAR	1.9–9.5	4.9	2.0
Heterogeneity of the canopy height (>0.2 m)	LiDAR	2.8–11.5	6.1	2.2
Mean canopy height (>5 m)	LiDAR	7.4–25.8	16.3	4.4
Mean canopy height (>2 m)	LiDAR	6.7–25.7	15.4	4.5
Mean canopy height (>0.2 m)	LiDAR	5.1–24.7	13.9	4.6
Plot elevation	DEM	1277–1969	1592	210

Variables were measured in the field, derived from LiDAR data or calculated using a digital elevation model (DEM). Range, mean and standard deviation values are presented for each variable. Cover variables and the contribution of each forest layer are expressed in percentage. Canopy heights and plot elevation are expressed in meters.

The principal component analysis conducted with the seven field-based understory descriptors extracted four components, absorbing 81% of the variance. The first component was negatively associated with forb cover, positively so with woody debris cover and to a lesser extent with needle litter cover. The second component was negatively associated with bare rock cover and positively associated with moss cover. The third component was negatively associated with fern cover. Finally, the fourth component was associated almost exclusively and positively with shrub cover (Table 2).

**Table 2:** Principal component analysis from the understory variables of the 50 forest plots, measured at 446 vegetation quadrats.

Understory structure	fPC1	fPC2	fPC3	fPC4
Shrub cover	0.077	-0.012	0.127	**0.897**
Forb cover	**-0.854**	0.168	0.312	-0.125
Fern cover	0.078	-0.029	**-0.961**	-0.156
Moss cover	-0.080	**0.747**	0.014	0.406
Needle litter cover	**0.668**	-0.286	0.449	-0.381
Woody debris cover	**0.759**	0.314	0.059	0.063
Bare rock cover	-0.083	**-0.838**	0.003	0.223
Eigenvalue	1.78	1.47	1.24	1.21
Explained variance (%)	25.38	21.01	17.74	17.26

Factor loadings for each variable after varimax rotation are shown for each extracted component (eigenvalue > 1). Factor loadings > |0.60| for each component (fPC1 to fPC4) are presented in bold type.

### LiDAR-based forest structure

The average canopy cover of mature trees was relatively high (70%), and even the more open plots had a minimum of 39% canopy cover (Table 1). When including the regeneration and shrub layers, minimum canopy cover was 62% and the average for all plots was 80% (Table 1). On average, tree layer was the main contributor to the vertical structure in all plots, followed by the herbaceous layer (Table 1). The relative contribution of the regeneration and shrub layers was low in all cases (Table 1). Height values for the tree layer (> 5 m) were less variable (i.e., lower heterogeneity) than those for the tree-plus-regeneration and tree-plus-regeneration-plus-shrub layers (canopy height heterogeneity > 2 m and canopy height heterogeneity > 0.2 m, respectively; Table 1). The principal component analysis performed with the thirteen LiDAR-based forest structure variables extracted three components that between them accounted for 85% of the overall variance. The first component was positively associated with canopy cover at different heights (5, 2 and 0.2 m) and with the relative contribution of the tree layer, and negatively correlated with the contribution of the herbaceous layer. This component can be interpreted as a gradient of forest openness, with closed canopy plots with a low herbaceous layer at one extreme and with more open plots with better developed herbaceous layers at the other (Table 3). The second component was positively associated with the contribution of the regeneration layer and the heterogeneity of canopy height at the three levels (Table 3). Thus, this component could be interpreted as a gradient of increasing complexity in plot vertical structure. Finally, the third component was positively correlated with mean canopy heights for the tree and regeneration layers. This component can be interpreted as a gradient in forest canopy height.

**Table 3:** Principal component loadings from the thirteen LiDAR-based forest structure variables, measured at 50 circular plots.

Forest structure	lPC1	lPC2	lPC3
Canopy cover at 5 m	**0.845**	-0.429	0.285
Canopy cover at 2 m	**0.944**	-0.224	0.120
Canopy cover at 0.2 m	**0.971**	-0.022	0.050
Contribution of the tree layer	**0.866**	-0.316	0.330
Contribution of the regeneration layer	-0.145	**0.738**	-0.504
Contribution of the shrub layer	-0.397	0.572	-0.202
Contribution of the herbaceous layer	**-0.857**	-0.385	-0.009
Heterogeneity of the canopy height (>5 m)	-0.040	**0.864**	0.173
Heterogeneity of the canopy height (>2 m)	-0.090	**0.929**	0.175
Heterogeneity of the canopy height (>0.2 m)	-0.234	**0.829**	0.456
Mean canopy height (>5 m)	0.124	0.228	**0.939**
Mean canopy height (>2 m)	0.165	0.040	**0.968**
Mean canopy height (>0.2 m)	**0.620**	-0.277	-0.062
Eigenvalue	4.70	3.78	2.59
Explained variance (%)	36.17	29.10	19.95

Loadings after varimax rotation for the three extracted components (eigenvalue > 1) are shown. Factor loadings > |0.60| for each component (lPC1, lPC2, lPC3) are presented in bold type.

### Relationships among field- and LiDAR-based components

Some components of the PCAs derived from field-based and LiDAR derived variables showed significant correlations (Table 4). Plots with more closed canopies had more cover of woody debris and needle litter, and less cover of forbs (Table 4). Plots that showed more complex forest vertical structure had more cover of shrubs and less cover of ferns. Finally, forest plots with tall canopies had more cover of moss and less rocks in the understory (Table 4).

**Table 4:** Product-moment pairwise correlations between field-based (fPC) and LiDAR-based components (lPC) extracted from the two principal component analyses.

PCA components	fPC1	fPC2	fPC3	fPC4
lPC1	0.343*	0.241	-0.074	-0.218
lPC2	0.141	0.271	-0.285*	0.348*
lPC3	-0.145	0.504**	-0.231	0.002

* *P* < 0.05

** *P* < 0.01

### Habitat modelling

The model fitted with structural variables derived from LiDAR included the first two components extracted from the PCA, elevation and the interaction between the second component (lPC2) and elevation (AIC = 233.5; Table 5). The model showed that the abundance of wood mouse decreased in plots with closed canopies and low contribution of the herbaceous layer (lPC1; Table 5; Fig 2), was negatively affected by elevation (Table 5), and increased with greater complexity of forest vertical structure (lPC2) at lower elevations (negative lPC2 × elevation interaction; Table 5). The best model based on field surveys (AIC = 246.5) only included the fourth component (fPC4) and elevation after model simplification, indicating that the abundance of wood mouse was positively influenced by the cover of shrubs in the plot and negatively influenced by elevation (Table 5). Simplification of the full model including elevation and predictors derived from LiDAR as well as field variables resulted in a model (AIC = 230.9) including two LiDAR-based predictors (lPC1, lPC2), elevation, the interaction between lPC2 and elevation, and one field-based (fPC4) predictor (Table 5). The simple model showed that the abundance of wood mouse was negatively influenced by elevation and positively associated with more open canopies and more herbaceous layer, with greater complexity of forest vertical structure at lower elevations, and more cover of shrubs (Table 5).

**Table 5:** Generalized linear models for wood mouse abundance in the Valsaín Scots pine forest.

Variable	Estimate	SE	Z	*P*	*D*^*2*^*(%)*
(a) LiDAR-based model:					26.9
Intercept	0.975	0.095	10.3	<0.001	
lPC1	-0.285	0.082	-3.5	<0.001	
lPC2	0.124	0.088	1.4	0.158	
lPC2 × Elevation	-0.343	0.091	-3.7	<0.001	
Elevation	-0.316	0.099	-3.2	0.001	
(b) Field-based model:					14.1
Intercept	1.112	0.083	13.4	<0.001	
fPC4	0.242	0.062	3.9	<0.001	
Elevation	-0.170	0.085	-2.0	0.046	
(c) Full model:					30.3
Intercept	0.962	0.096	10.0	<0.001	
lPC1	-0.255	0.083	-3.1	0.002	
lPC2	0.069	0.093	0.7	0.460	
lPC2 × Elevation	-0.329	0.093	-3.5	<0.001	
Elevation	-0.309	0.102	-3.0	0.002	
fPC4	0.166	0.074	2.2	0.024	

Estimates and standard errors (SE) from the best generalized linear model, using elevation and habitat predictors derived from (a) LiDAR, (b) field, or (c) both groups of variables. The amount of deviance accounted for the models is represented by D^2^.

### Variance partitioning

**Fig 3:**
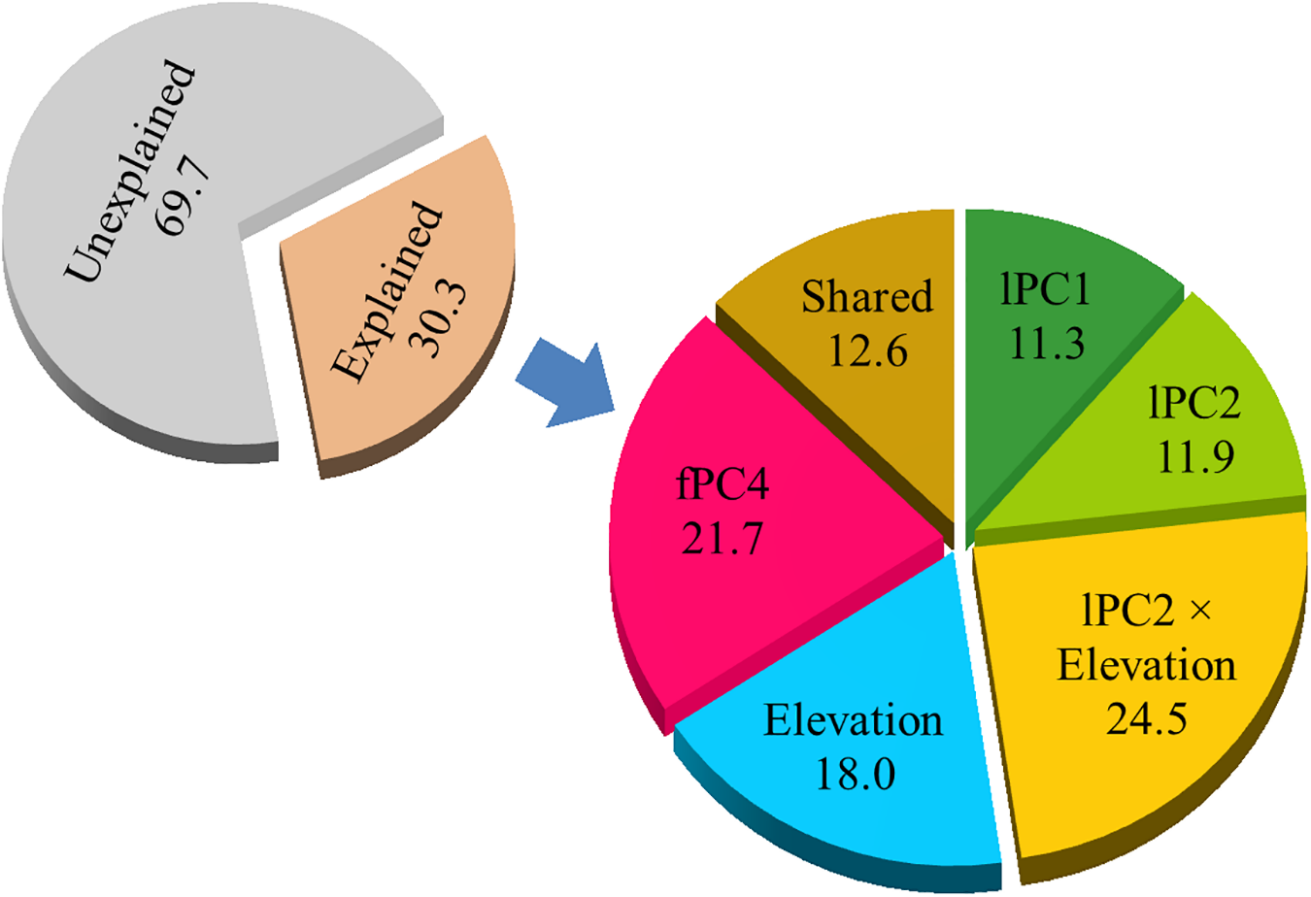
Variance partitioning from linear model using elevation, field- and LiDAR derived variables for wood mouse abundance in Valsaín forest. Percentage of variance explained by the combined model (small pie chart), and percentage of that variation explained by elevation, LiDAR- and field-based variables, and their shared contribution to the model (large pie chart). LiDAR and field-based variables correspond to those in the best simple model with both sets of variables (see Table 5).

Hierarchical partitioning of variance showed that the four variables in the model and the interaction accounted for 87.4% of the explained variance, and that the remainder 12.6% was shared by the predictors (Fig 3). The proportion of explained variance was mainly accounted by the second LiDAR-based component (lPC2) and its interaction with elevation, followed by the field-based variable (fPC4), elevation and the first LiDAR-based component (lPC1) (Fig 3).

## Discussion

LiDAR has recently arisen as a useful technology to model wildlife-habitat structure relationships [9, 10]. However, the majority of studies using this technology have focused on flying animals, mostly vertebrates, whereas studies on non-flying fauna are still lacking [9, 10]. Our results modelling the habitat preferences of wood mouse in a Mediterranean pine high mountain forest indicate that LiDAR derived variables were better predictors than field-based variables. The model combining both data sets slightly improved the predictive power of the model. Our results suggest that LiDAR is a promising technology for further exploring habitat preferences by small mammal communities.

### LiDAR and field derived models

The model based on LiDAR variables accounted for almost double of the variation in wood mouse abundance compared to the model based on field data. This result set light on the potential of using airborne LiDAR data to model habitat preferences of small mammals in forested habitats. LiDAR derived variables were summarized into three components that sorted forest patches in gradients of canopy openness, complexity of plot vertical structure and canopy height. Elevation and two of the structural features were associated with the abundance of wood mouse, particularly the complexity of vertical structure. Elevation has a negative effect on the diversity and abundance of small mammal communities in Mediterranean mountains [38, 43], as has been previously described for the wood mouse in central Spain [21]. In addition, elevation modulated the response of wood mouse to the vertical complexity of forest structure.

The complexity of vertical structure has been consistently found as a key habitat component for flying vertebrates in LiDAR studies due to the intrinsic ecology of these organisms [44–47]. LiDAR studies in terrestrial species are still limited and have shown that canopy openness was important for terrestrial species, although the importance of the vertical component has not previously been identified affecting preferences in terrestrial species [9, 10]. In our study area, forest patches with greater complexity in vertical structure corresponded to patches including mature trees as well as regeneration and shrub layers (Fig 2A). Forest structure in these patches is the result of management practices through successive partial harvests leaving older trees as seed sources and protection for regeneration [33]. Harvests create gaps promoting regeneration and the presence and cover of understory vegetation [48, 49], that provides food and shelter for terrestrial species [28, 50]. Interestingly, our results agree with previous findings relating forest structural characteristics to small mammal communities using traditional field measurements. Small mammals were more abundant in regeneration stands where patches retain features of mature stands together with greater understory cover, typical of early successional stages after disturbance [24, 27, 51].

The abundance of wood mouse was also negatively influenced by closer canopies. Patches with close canopies in the forest of Valsaín are characteristic of relatively even-aged stands before initiating successive harvests to foster regeneration [33]. These structurally simple patches mostly show developed canopy layers with few gaps and nearly no regeneration and shrub layers (Fig 2B).

The model using structural characteristics of the understory measured in the field indicated that patches with greater cover of shrubs positively influenced the abundance of wood mouse and that the abundance decreased with elevation. This expected result is in line with many studies that highlight the relevance of shrub cover for rodent mammals [22, 34, 52]; shrubs offer safe sites for small mammals reducing predation risk and increasing foraging efficiency. Because shrubs provide crucial resources for small mammals (i.e., shelter, food, nesting sites), and are relatively scarce in this pine forest (as in other temperate pine forests), they could be considered as ‘keystone structures’ (*sensu* [2]; see review in [53]) that should be kept during silvicultural treatments. On the other hand, other components of the ground layer were not significant for wood mouse in this pine forest. For example, woody debris and needle litter may increase the availability of invertebrates and fungi, which are potential food resources for small mammals [54–56]. However, woody debris and needle litter (fPC1) tended to increase in forest patches with closed canopies (lPC1; *r* = 0.34, *P* = 0.015), where the abundance of wood mouse decreased. Our results suggest that some features of the ground layer may not be important if the key structure (i.e., shrub cover) is not present in the forest patch.

### Combined model and variance partitioning

The model predicting the abundance of wood mouse using LiDAR and field derived variables slightly increased the fraction of variability explained (30%) compared to the LiDAR model (27%). The model was similar to the LiDAR model and included one field derived environmental variable, indicating that the abundance was positively associated with greater complexity of forest vertical structure at lower elevations and greater cover of shrubs, and negatively influenced by elevation and close canopies with low herbaceous layer.

Variance partitioning showed that LiDAR-derived structural components, together with elevation, explained most of the variation, although shrub cover still explained an important fraction of the variance, and there was a 13% of shared variance between variables (Fig 3). This result further supports the importance of shrubs as a key structure in this pine forest with low and sparse cover of shrubs (9% on average). Variations in shrub cover among forest patches were mainly captured by the second component of the PCA derived from LiDAR data (Table 4), although shrub layer contributed less to that component than other structural variables. Therefore, field measurement of shrub cover seems to be an efficient, although more time-consuming, approach to estimate an important habitat feature for wood mouse in this pine forest. However, the predictive power of LiDAR derived variables was much greater than that of field-based measurements, and only slightly improved when combining both data sets. Consequently, LiDAR derived variables were good surrogates of environmental characteristics favoring the abundance of wood mouse. Our LiDAR metrics represented structural features of the forest patch, such as the presence and cover of shrubs, as well as other characteristics likely including time since perturbation, food availability and predation risk.

However, our results should be taken with caution due to the time frame of the study. It is known that habitat preferences in small mammals can vary with time as a consequence of fluctuations in population density (see e.g. [56, 57]). Thus, it would be desirable to conduct long-term studies to minimize potential biases due to changes in the population dynamics of wood mouse.

### Management implications

Small mammals play important roles in diverse ecological processes in forest ecosystems [58]. In order to counteract negative impacts on wood mouse populations, forest managers should avoid the destruction of understory vegetation during silvicultural practices. Wood mouse benefited from the environmental conditions generated by the opening of small gaps through partial harvesting to promote natural pine regeneration that favor the development of understory vegetation and increasing the heterogeneity of the vertical structure. Because long regeneration periods increase stand structural diversity throughout the whole rotation period [33, 59], forest management should be oriented to long rotation periods. In addition, dense stands of even-aged trees with close canopies lacking understory vegetation should be avoided.

### Conclusions

Wood mouse was influenced by forest vegetation structure and elevation. Structural variables derived from airborne LiDAR were able to capture variations in the shrub layer and likely correlated with other unmeasured abiotic and biotic variables that are relevant to wood mouse, thus showing greater predictive performance than field-based variables. Airborne LiDAR provides information on forest structure at fine scales, relevant to biodiversity, and across large areas at affordable costs that cannot be reliably collected in the field.

Therefore, LiDAR information allows the upscaling and mapping of biodiversity and ecosystem processes to broad spatial extents. We recommend the use of LiDAR data (e.g., LiDAR data derived from national inventories) to conduct more research on wildlife-habitat relationships, particularly on less well-studied terrestrial species such as small rodent communities. This will help expand our knowledge on biodiversity distribution at various spatial scales and assist conservation managers in decision-making.

## Supporting information

S1 Dataset Analysis file for wood mouse abundance in the Valsaín forest. (XLSX)
